# Energy metabolic shift contributes to the phenotype modulation of maturation stage ameloblasts

**DOI:** 10.3389/fphys.2022.1062042

**Published:** 2022-11-29

**Authors:** Haruno Arai, Akira Inaba, Shojiro Ikezaki, Mika Kumakami-Sakano, Marii Azumane, Hayato Ohshima, Kazumasa Morikawa, Hidemitsu Harada, Keishi Otsu

**Affiliations:** ^1^ Division of Developmental Biology and Regenerative Medicine, Department of Anatomy, Iwate Medical University, Yahaba, Japan; ^2^ Division of Pediatric and Special Care Dentistry, Department of Oral Health Science, School of Dentistry, Iwate Medical University, Morioka, Japan; ^3^ Division of Oral and Maxillofacial Surgery, Department of Reconstructive Oral and Maxillofacial Surgery, Iwate Medical University, Morioka, Japan; ^4^ Division of Anatomy and Cell Biology of the Hard Tissue, Department of Tissue Regeneration and Reconstruction, Niigata University Graduate School of Medical and Dental Sciences, Niigata, Japan

**Keywords:** tooth, enamel, ameloblast, energy metabolism, OxPhos, glycolysis, hypoxia, mineralization

## Abstract

Maturation stage ameloblasts (M-ABs) are responsible for terminal enamel mineralization in teeth and undergo characteristic cyclic changes in both morphology and function between ruffle-ended ameloblasts (RA) and smooth-ended ameloblasts (SA). Energy metabolism has recently emerged as a potential regulator of cell differentiation and fate decisions; however, its implication in M-ABs remains unclear. To elucidate the relationship between M-ABs and energy metabolism, we examined the expression pattern of energy metabolic enzymes in M-ABs of mouse incisors. Further, using the HAT7 cell line with M-AB characteristics, we designed experiments to induce an energy metabolic shift by changes in oxygen concentration. We revealed that RA preferentially utilizes oxidative phosphorylation, whereas SA depends on glycolysis-dominant energy metabolism in mouse incisors. In HAT7 cells, hypoxia induced an energy metabolic shift toward a more glycolytic-dominant state, and the energy metabolic shift reduced alkaline phosphatase (ALP) activity and calcium transport and deposition with a change in calcium-related gene expression, implying a phenotype shift from RA to SA. Taken together, these results indicate that the energy metabolic state is an important determinant of the RA/SA phenotype in M-ABs. This study sheds light on the biological significance of energy metabolism in governing M-ABs, providing a novel molecular basis for understanding enamel mineralization and elucidating the pathogenesis of enamel hypomineralization.

## Introduction

**FIGURE 1:**
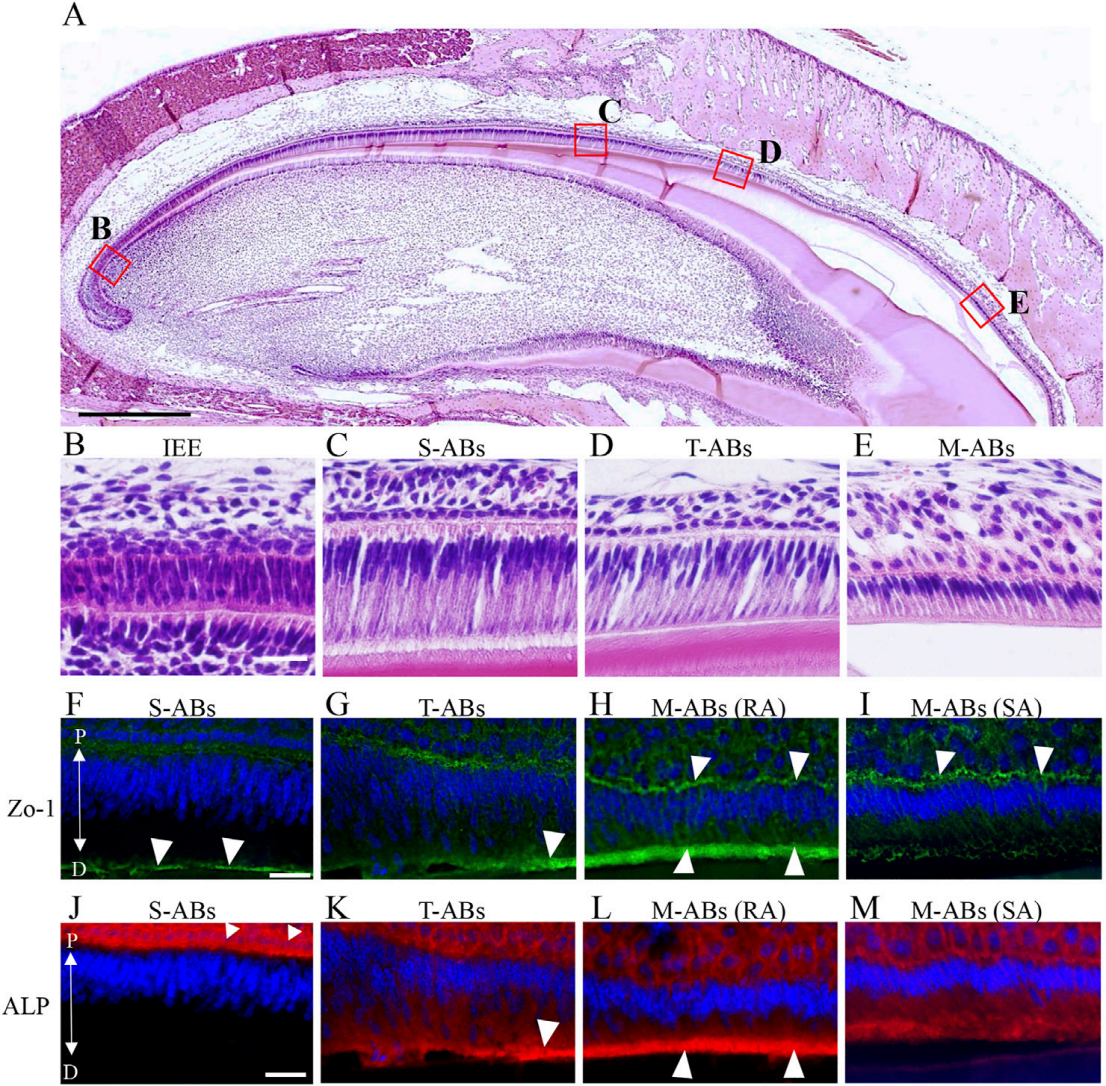
Differential expression of Zo-1 and ALP during amelogenesis in the maxillary incisor. **(A)** Low magnification image of H&E-stained sections of mouse maxillary incisors. The boxed areas in **(A)** are magnified in **(B–E)**. **(B)** Inner enamel epithelium cells. **(C)** Secretory stage ameloblasts. **(D)** Transition stage ameloblasts **(E)** Maturation stage ameloblasts. **(F–I)** Zo-1 immunostaining of mouse maxillary incisor ameloblasts. **(J–M)** ALP staining of mouse maxillary incisor ameloblasts. The nucleus is stained with DAPI (blue). S-ABs, secretory stage ameloblasts; T-ABs, transition stage ameloblasts; M-ABs, maturation stage ameloblasts; RA, ruffle-ended ameloblasts; SA, smooth-ended ameloblasts; P, proximal; D, distal. Scale bars: 500 μm **(A)**; 20 μm **(B–M)**.

Enamel is the most highly mineralized tissue in the vertebrate body and is composed of substituted hydroxyapatite, primarily calcium and inorganic phosphate. Ameloblasts, which are responsible for enamel formation, are oral epithelial cells of ectodermal origin. The proliferating inner enamel epithelium (IEEs) differentiates into secretory stage ameloblasts (S-AMs), and they differentiate into maturation stage ameloblasts (M-ABs) through transition stage ameloblasts (T-ABs) (Figures 1A–E). S-AMs secrete enamel matrix proteins, which form the base of enamel and contribute to the initial calcification, while M-ABs modulate enamel mineralization by transporting minerals, controlling pH, and modulating protein degradation and absorption (Nanci, 2008; Bartlett, 2013).

During the maturation stage, ameloblasts change their morphology in a unique series of modulations (cyclical changes) between a ruffle-ended (RA) appearance and a smooth-ended (SA) appearance in coordinated groups, appearing as bands of similar morphology (Warshawsky and Smith, 1974; Reith and Boyde, 1981). RA cells are characterized by distinct distal striated or ruffled borders (Reith and Boyde, 1979). In contrast, SA cells exhibit a complete absence of the distal ruffled border (Sasaki et al., 1987). RA has a greater capacity to transport ions into and away from the enamel matrix and to absorb the enamel matrix protein debris. SA with incomplete junctional complexes may engage in the paracellular movement of fluids and ions, which may contribute to the neutralization of pH in the enamel matrix (Lacruz, 2017). SA appear at ∼8.5 h intervals in rat incisors, and these ameloblasts change into RA cells after 2 h, reforming their characteristic features at the distal border (Smith et al., 1987). Thus, cyclic RA-SA modulation is crucial for normal enamel mineralization. However, the regulatory mechanisms and determinants that distinguish RA from SA are not yet understood.

A close relationship between energy metabolism, cellular differentiation, and fate decisions has emerged in recent years. Early embryos are dependent on oxidative phosphorylation (OXPHOS). As developmental stages progress, they utilize the glycolytic system to synthesize ATP, which peaks after implantation and slowly declines as oxidative metabolism is reinitiated by vascularization (Folmes et al., 2012). Human ES and iPS cells differ in their energy metabolism state between the naïve type, which is close to the internal cell mass before implantation, and the primed type, which resembles pluripotency in the epiblast after implantation (Tsogtbaatar et al., 2020). Recently, we reported that, in ameloblasts, slowly dividing dental epithelial stem cells are glycolytic-dominated, while rapidly dividing transient amplifying (TA) cells are OXPHOS-dominated in their energy metabolism (Otsu et al., 2021), indicating the implication of energy metabolism in the cell fate decision of ameloblasts. Based on this, we hypothesized that energy metabolism is involved in RA-SA modulation in M-ABs.

In this study, we explored the change of energy metabolic characteristics in M-ABs immunohistochemically. To elucidate the effect of an energy metabolic shift on M-ABs, we utilized the change of oxygen concentration. We found that RA and SA have distinct characteristics of energy metabolism and that metabolic shift is a potential regulator of RA-SA modulation of M-ABs. Our study proposes a novel perspective on enamel research and attempts to elucidate the pathogenesis of enamel hypomineralization.

## Materials and methods

### Animals and preparation of tissues

All animal experiments complied with the guidelines of the Ministry of Education, Culture, Sports, Science and Technology, the Ministry of Environment, and the Science Council of Japan, and were carried out in accordance with the Act on Welfare and Management of Animals. The experimental protocol was approved by the Institutional Animal Care and Use Committee (approval no. 01-007). For hematoxylin and eosin (H&E) staining and immunostaining, ddY male mice (Japan SLC) mouse jaws were fixed in 4% paraformaldehyde (PFA) and decalcified using Osteosoft (#101728, Merck, Darmstadt, Germany) and paraffin-embedded thin tissue sections (thickness, 6-7 μm) were used. Kawamoto’s film method was used to detect the activity of alkaline phosphatase (ALP) in mouse incisors (Kawamoto, 2003). Briefly, the jaws were taken from ddYmice, snap-frozen directly, soaked in hexane with dry ice, and embedded in an embedding medium. The samples were sectioned at 10 μm thickness using a cryostat. Sections were moved to a container filled with the appropriate amount of 100% ethanol, fixed with 4% paraformaldehyde (PFA) for 5 min, and washed. The specimens were stained with the ImmPACT Vector Red Alkaline Phosphatase Substrate kit (#SK-5105, Vector, Burlingame, CA, United States) according to the manufacturer’s protocol. For analysis of cytochrome oxidase (CO) activity in ameloblasts using transmission electron microscopy, the animals (30-day-old Wistar rats) were anesthetized and perfused through the ascending aorta with physiological saline, followed by 2.5% glutaraldehyde in 0.1 M phosphate buffer (pH 7.4) at 4°C for 10 min. The removed maxillae were immersed in the same fixative at 4°C for 2 h before decalcification in 5% ethylenediaminetetraacetic acid (EDTA) at 4°C for 3 weeks, then sagittally sectioned (90-μm sections) using a vibratome (Brunswick, St. Louis, MO, United States). At least three animals were studied for each experiment.

### Cell culture

The ameloblast cell line HAT7 was established from rat incisors and cultured as previously described (Kawano et al., 2002). The cells were maintained in Dulbecco’s modified Eagle’s medium (DMEM/F12) (#11330-032; Life Technologies, Inc., Grand Island, NY, United States) supplemented with 10% fetal bovine serum (#12483-020, Thermo Scientific, Waltham, MA, United States) and 1% penicillin-streptomycin (#15140; Thermo Fisher Scientific). To induce hypoxia, the cells were cultured in hypoxic chambers (MCO-5M, PHCbi, Tokyo, Japan) with 5% O_2_, 5% CO_2_, and 90% N_2_. Nitrogen gas was supplied to the chambers to induce a controlled reduced percentage of oxygen. For normoxia, the cells were cultured in incubators at 5% CO_2_ and 21% O_2_. Apoptotic cells were determined by Annexin V staining (#A13199, Thermo Fisher Scientific) according to the manufacturer’s instructions. As a positive control of apoptosis induction, the cells were treated with mitomycin C (#M4287, Sigma-Aldrich, St Louis, MO, United States, 50 μM) for 6 h.

### Alizarin red staining

For alizarin red staining for calcium deposition, HAT7 cells were cultured in 24-well plastic plates coated with collagen type I (#638-00781, Nitta Gelatin Co., Osaka, Japan) at confluence in calcification induction medium; DMEM/F12 supplemented with 10% FBS, dexamethasone (10 nM), CaCl_2_ (final concentration 2.1 mM) for 7 days under normoxia (21% O_2_) or hypoxia (5% O_2_), or for 5 days with UK-5099 (#S5317, Selleckchem, Randnor, PA, United States). The culture supernatant in the wells was removed, and the cells were washed with PBS, fixed with 4% PFA, and then washed three times with distilled water. Next, a 1.0% Alizarin Red S (#A5533, Sigma-Aldrich) stain was added, and the mixture was allowed to stand at room temperature for 30 min. The cells were then washed three times with PBS. The collagen gels with the cells were placed on the prepared slide and then dried at 37°C for 1 h. After drying, the gels were observed.

### Alkaline phosphatase staining

HAT7 cells were cultured in 24-well plastic plates at confluence and cultured under normoxia or hypoxia for 48 h, or with UK-5099 for 48 h. The culture supernatant was removed, and the cells were washed with PBS and then fixed in wells with 4% PFA for 10 min at room temperature (RT). Thereafter, the fixative solution was removed, and the cells were washed three times with PBS. Subsequently, the substrate (ImmPACT Vector Red Alkaline Phosphatase Substrate) was added and reacted at 37°C for 30 min. Finally, after washing three times with PBS, the staining was observed.

### *In vitro* calcium transport assay

HAT7 cells were grown on permeable polyester Transwell culture inserts with a 0.4-μm pore size (#353095 Corning Inc., Corning, NY, USA) at confluence. The medium in both the upper and lower chambers was then changed to an induction medium, and the cells were cultured under normoxia or hypoxia. After 24 h, the medium in the lower chamber was replaced with Ca^2+^-free DMEM (#21068028, Thermo Fisher Scientific, Waltham, MA, United States), and the cells were continuously cultured. At various time intervals (6, 12, 24, and 48 h after medium change), 50 μl aliquots of media from the lower chamber were collected into 1.5 ml Eppendorf tubes. The amount of Ca^2+^ in the media was evaluated with an Amplite^TM^ Fluorimetric Calcium Quantitation Kit (#36360, AAT Bioquest, CA, United States) by measuring the fluorescence intensity using a multi-mode microplate reader (SpectraMax M2, Molecular Devices, CA, United States) with excitation at 540 nm and emission at 590 nm, according to the manufacturer’s protocol. Increases in the amount of Ca^2+^ transferred through the cell layer from the upper chamber to the lower chamber indicate increased Ca^2+^ transport across the cells. After reaching confluence, the HAT7 cells on Transwell filters were fixed in 4% PFA, and the filters were removed from the plastic inserts and cut into strips. Some strips were processed for paraffin cross-sections, dewaxed, and stained with H&E. To obtain an en-face view, other strips were transferred to 24-well plates, rinsed in PBS containing Triton X-100 (0.01% v/v), immunoreacted with primary antibodies, followed by incubation with secondary fluorescent antibodies, and then observed.

### ATP measurement in culture cells

HAT7 cells were cultured in 24-well plastic plates at confluence and then maintained under normoxia or hypoxia for 48 h. The cells were harvested using the extraction solution provided in the Intracellular ATP assay kit (#IC2-100, Toyo Ink Group, Tokyo, Japan). Luciferin substrate and luciferase enzyme were added, and bioluminescence was assessed using a multi-mode microplate reader according to the manufacturer’s instructions. Cellular ATP levels were evaluated and expressed as the ratio of hypoxic to normoxic conditions.

### Staining of mitochondria with probes

HAT7 cells were cultured in 96-well plastic plates at confluence and then maintained under normoxia or hypoxia for 48 h. Mito Tracker Orange CMXRos (500 nM, #M7510, Thermo Fisher Scientific) or JC-1 (2 μmol/l, #MT09, Dojindo, Kumamoto, Japan) was added to the cells and incubated for 60 min at 37°C. The cells were washed 2 × with culture media, and fluorescence images were obtained using a fluorescence microscope (BX51, IX71, Olympus, Tokyo, Japan). JC-1 green/red fluorescence ratios were calculated and analyzed statistically.

### Lactate assay

HAT7 cells were cultured in 24-well plastic plates at confluence and then maintained under normoxia or hypoxia for 48 h. The supernatant was collected, and the released lactate level in the medium was measured using a Lactate Assay Kit-WST (#L256, Dojindo) following the manufacturer’s instructions.

### Immunohistochemistry and immunofluorescence

Immunohistochemical (IHC) and immunofluorescent (IF) staining were performed as previously described (Otsu et al., 2011). After blocking, the samples were incubated with the following antibodies (1:100): PDH (MA5-14805, Thermo Fisher Scientific), Zo-1 (sc-33725, Santa Cruz, Dallas, TX, USA) and LDH (ab52488, Abcam). DAPI (300 nM; D1306), Hoechst 33,342 (#R37605), Alexa Fluor 488 (1:500), and Alexa Fluor 546 (1:500) secondary antibodies were purchased from Thermo Fisher Scientific. Images were obtained using a fluorescence microscope (BX51, IX71; Olympus) or laser-scanning confocal microscope (C1si, Nikon). Image analyses were performed using ImageJ or software provided by the microscope. Fluorescence intensity was quantitated in at least five randomly chosen fields of view using the same threshold. Appropriate positive and negative controls were used for each experiment.

### RT-PCR

Total RNA was extracted using the RNeasy Mini Kit (#74104, Qiagen, Hilden, Germany). Reverse transcription of total RNA was performed using the PrimeScript RT reagent kit (#RR037A, Takara Bio, Otsu, Japan). Quantitative analysis of gene expression was performed by qRT-PCR using the TB Green Fast qPCR Mix (#RR430A, Takara Bio, Otsu, Japan) and oligonucleotide primers specific for the target sequences (Table 1) on a Thermal Cycler Dice (Takara Bio, Otsu, Japan) according to the manufacturer’s protocol. The specificity of the PCR was confirmed by the appearance of a single band of PCR product in 2% agarose gel stained with ethidium bromide. The target gene expression levels were normalized to the corresponding levels of GAPDH mRNA. Gene expression levels were calculated relative to the values in control cultures using the comparative Ct (2^−ΔΔCT^) method. The experiments were performed in triplicates.

**TABLE 1:** List of PCR primer used in this study.

mRNA	Orientation	Sequence (5’-> 3′)
Gapdh	Forward	GGC​ACA​GTC​AAG​GCT​GAG​AAT​G
	Reverse	ATG​GTG​GTG​AAG​ACG​CCA​GTA
ZO-1	Forward	CGG​AAA​TGT​GTA​AAT​CAC​CTG​GAA
	Reverse	CAT​GCG​TCC​TGA​ACA​CAT​CAA​AC
Wdr72	Forward	GAA​CTC​GGC​AAA​CTT​CCA​AGA​TAC​A
	Reverse	GGA​GCA​CAC​CTT​CGC​TAT​CCA
Klk-4	Forward	TTT​TGC​CAA​CGA​CCT​CAT​GCT​C
	Reverse	AAC​CAG​AAA​CTA​GGC​AGG​TAT​CCC
Stim1	Forward	CTC​CAG​GGC​TCC​ATT​CAG​ACA
	Reverse	ACA​GCT​TTG​GCA​TCT​ACT​CAT​CCT​C
Orai1	Forward	TCA​AAG​CCT​CCA​GCC​GAA​C
	Reverse	GAT​GAG​TAA​CCC​TGG​CGG​GTA​GT
Cnmm4	Forward	AGA​TGG​CGG​CTT​TCA​ACG​A
	Reverse	GCA​TGC​CGC​ACC​TAC​AGA​GA
Slc24a4	Forward	TAG​CTT​GGC​ACA​TCC​CAT​GAA​C
	Reverse	TTG​CCC​AGA​AAA​CAG​GAG​GAA​C
Odam	Forward	CGA​TTG​CTC​CAC​TGC​TTC​CA
	Reverse	ACG​CCA​AGG​TAC​CAT​CTC​ATC​TTC
Cldn1	Forward	AAG​GCT​TTC​GGT​TGT​GAG​TCA​G
	Reverse	AGG​CAG​AAG​GAT​GTT​TGT​GTG​G
Cldn2	Forward	ATT​CGA​GTC​ATC​GCC​CAT​CAG
	Reverse	CCA​GGC​AGA​AGT​TCA​CCA​ATC​A
Cldn4	Forward	ACG​AGA​CCG​TCA​AGG​CCA​AG
	Reverse	GTC​CAG​GAC​ACA​GGC​ACC​ATA​A
Cldn8	Forward	TTA​TGC​ACA​CTG​CTT​CAA​TTG​TTC​C
	Reverse	GAA​ATC​GCA​GCT​TAA​ACC​AAC​AGT​C
Cldn12	Forward	ATG​TGA​GAT​GGC​GCA​GCA​AG
	Reverse	ACA​GGG​CGT​ATG​TAC​ACG​CAG​A
Cldn19	Forward	GGC​AGG​TGC​AAT​GCA​AAC​TCT​A
	Reverse	CTG​AGC​ACC​ATG​GCC​ACA​A
Glut1	Forward	ATA​GTC​ACA​GCA​CGT​CCA​TTC
	Reverse	TGT​AGA​ACT​CCT​CAA​TTA​CCT​TCT​G
Hk2	Forward	GAA​CAG​CCT​AGA​CCA​GAG​CAT​CC
	Reverse	ACG​GCA​ACC​ACA​TCC​AGG​TC
PDK1	Forward	TCA​ACT​ACA​TGT​ACT​CAA​CTG​CAC
	Reverse	ACT​CCG​TTG​ACA​GAG​CCT​TAA​TA
PDK2	Forward	CCA​TGA​AGC​AGT​TTC​TAG​ACT​TCG
	Reverse	CAG​ACT​CTG​GAC​ATA​CCA​GCT​C
PDK3	Forward	TGT​GAA​CAG​TAT​TAC​CTG​GTA​GCT​C
	Reverse	CTG​TTG​CTC​TCA​TCG​AGT​TCT​TG
LDHA	Forward	GTG​CAC​TAA​GCG​GTC​CCA​AA
	Reverse	GCA​AGC​TCA​TCA​GCC​AAG​TC

### Transmission electron microscopy

Analysis of cytochrome oxidase activity in ameloblasts using transmission electron microscopy has been described previously (Ohshima et al., 1998). The sections (90-μm) were incubated for the demonstration of CO activity according to Seligman et al. (Seligman et al., 1968): preincubation in 0.1 M phosphate buffer (pH 7.4) with 1 mg/ml catalase for 10 min at 37°C, and incubation immediately in a medium consisting of 0.1 M phosphate buffer (pH 7.4) containing 1 mg/ml 3,38-diaminobenzidine (DAB) tetrahydrochloride, 0.1 mg/ml catalase, 1 mg/ml cytochrome c (horse heart, type III, Sigma Chemical Co., St Louis, MO), 85 mg/ml sucrose at 37°C for 1 h. After washing in the cold phosphate buffer, the incubated sections were post-fixed in 1% osmium tetroxide containing 1.5% potassium ferrocyanide for 1 h, and then dehydrated through a graded series of ethanol, and embedded in Epon 812. Ultrathin sections (70 nm) were prepared using a Reichert Ultracut-N ultramicrotome (Reichert-Nissei, Tokyo, Japan) with a diamond knife. Samples were examined under a Hitachi H-7000 transmission electron microscope (Hitachi Co. Ltd., Tokyo, Japan) without staining.

### Statistical analyses

All data are reported as the mean ± SD. Differences were considered statistically significant if *p* < 0.05 by Student’s *t*-test. * denotes *p* < 0.05.

## Results

### Identification of differential developmental stage of ameloblasts

First, we investigated differences in the distribution of tight junction proteins and ALP activity in each differentiation stage of ameloblasts from S-ABs to early M-ABs in mouse incisors. Immunofluorescence showed that a punctiform expression of Zo-1 was observed at the distal end of S-ABs (Figure 1F, arrowheads). The expression in T-ABs gradually became stronger toward the incisal end (Figure 1G, arrowhead). Distinct expression of Zo-1 was observed at both the distal and proximal ends of the RA (Figure 1H, arrowheads) but only at the proximal end of the SA (Figure 1I, arrowheads). ALP staining revealed that S-ABs did not show any ALP activity, whereas the strong activity was observed in the stratum intermedium (Figure 1J arrowheads). The activity gradually increased at the distal end of T-ABs (Figure 1K, arrowhead). Strong ALP activity was observed at the distal end of the RA (Figure 1L, arrowheads), but it was weak in the SA (Figure 1M).

### Energy metabolic shift occurs during ameloblasts differentiation *in vivo*

**FIGURE 2:**
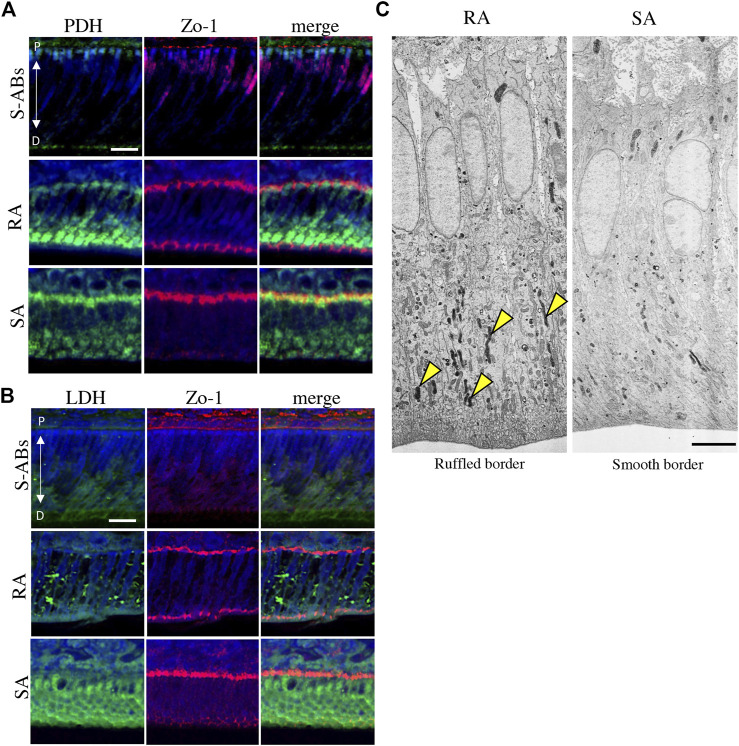
Energy metabolic state during ameloblasts differentiation *in vivo*. Double immunostaining for Zo-1 and PDH **(A)** and LDH **(B)** in P10 mouse maxillary incisors. Nuclei were stained with DAPI (blue). **(C)** Electron microscopic images of cytochrome oxidase (CO) activity in rat ruffle-ended (left) and smooth-ended (right) ameloblasts. Arrowheads indicate CO-positive mitochondria. Scale bars:20 µm **(A,B)** and 5 µm **(C)**.

We further examined the difference in the energy metabolic state between S-ABs and early M-ABs. The expression of pyruvate dehydrogenase (PDH), which aerobically catalyzes the conversion of pyruvate to acetyl-CoA for use in mitochondrial metabolism (Harris et al., 2002), gradually increased from S-ABs to RA, and a distinct expression was observed at the distal end of RA. In contrast, expression in SA was weaker than that in RA (Figure 2A). The expression of LDH, which catalyzes the conversion of pyruvate to lactate during glycolysis (Doherty and Cleveland, 2013), was weak in S-ABs. The expression of LDH in RA cells exhibited a punctate pattern in the cytoplasm, whereas it became stronger throughout the cytoplasm in SA cells (Figure 2B).

The well-developed mitochondrial apparatus has been implicated as an important indicator of substantial energy-generating potential, permitting, for example, active ion transport function (Garant and Nalbandian, 1968; Hubbard, 2000). To compare the functional activity of mitochondria in RA and SA *in vivo*, the activity of CO, a membrane-bound mitochondrial enzyme involved in OXPHOS, was analyzed using transmission electron microscopy (TEM). A large population of mitochondria in the distal cytoplasm was positive for CO (Figure 2C, arrowheads), whereas mitochondria in the distal cytoplasm displayed diversity in the proportion of CO activity in SA, suggesting that the activity of mitochondria in RA was higher than that in SA. Together, these results indicate that during differentiation, ameloblasts change their energy metabolic status and suggest that RA preferentially utilizes OXPHOS in mitochondria with high oxygen consumption, whereas SA undergoes a metabolic switch toward glycolysis-dominant energy metabolism.

### Energy metabolic shift by oxygen in HAT7 cells

**FIGURE 3:**
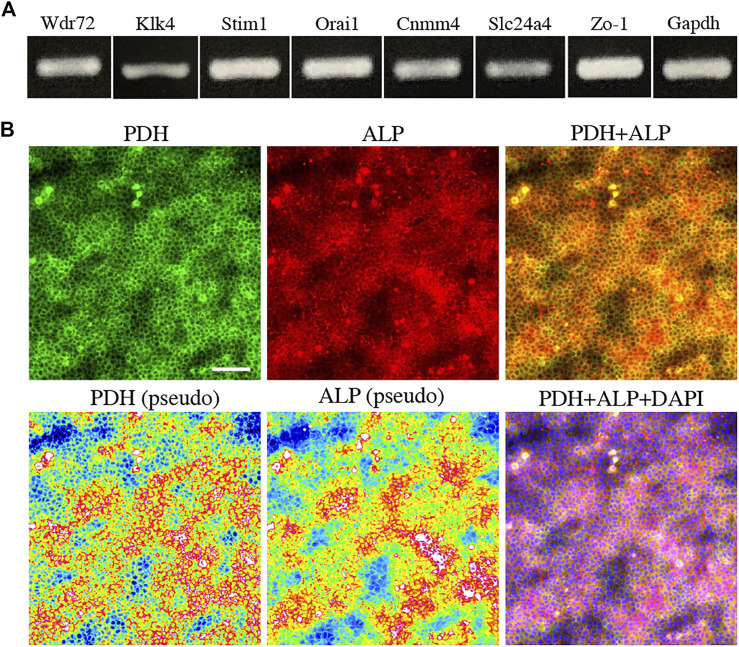
The expression of marker for maturation stage ameloblasts and energy metabolism in HAT7 cells. **(A)** The expression of maturation stage ameloblasts marker in HAT7 cells, as determined by RT-PCR. **(B)** Double staining of PDH and ALP in HAT7 cells. The bottom left and bottom middle images are the pseudo-color images of PDH and ALP, respectively. The nucleus is stained with DAPI (blue). Scale bars: 50 μm.

To further elucidate the relationship between M-ABs and energy metabolic states, we performed *in vitro* experiments using the ameloblast cell line HAT7, which has been shown to possess some of characteristics of M-ABs (Bori et al., 2016). First, we validated the expression of the M-AB marker in HAT7 cells. PCR analysis revealed that HAT7 cells expressed Wdr72, Klk4, Stim1, Orai1, Cnmm4, Slc24a4, and Zo-1 (Figure 3A). Furthermore, HAT7 cells expressed PDH, and the expression pattern was consistent with that of ALP activity (Figure 3B), consistent with *in vivo* results (Figures 1L,M, 2A).

**FIGURE 4:**
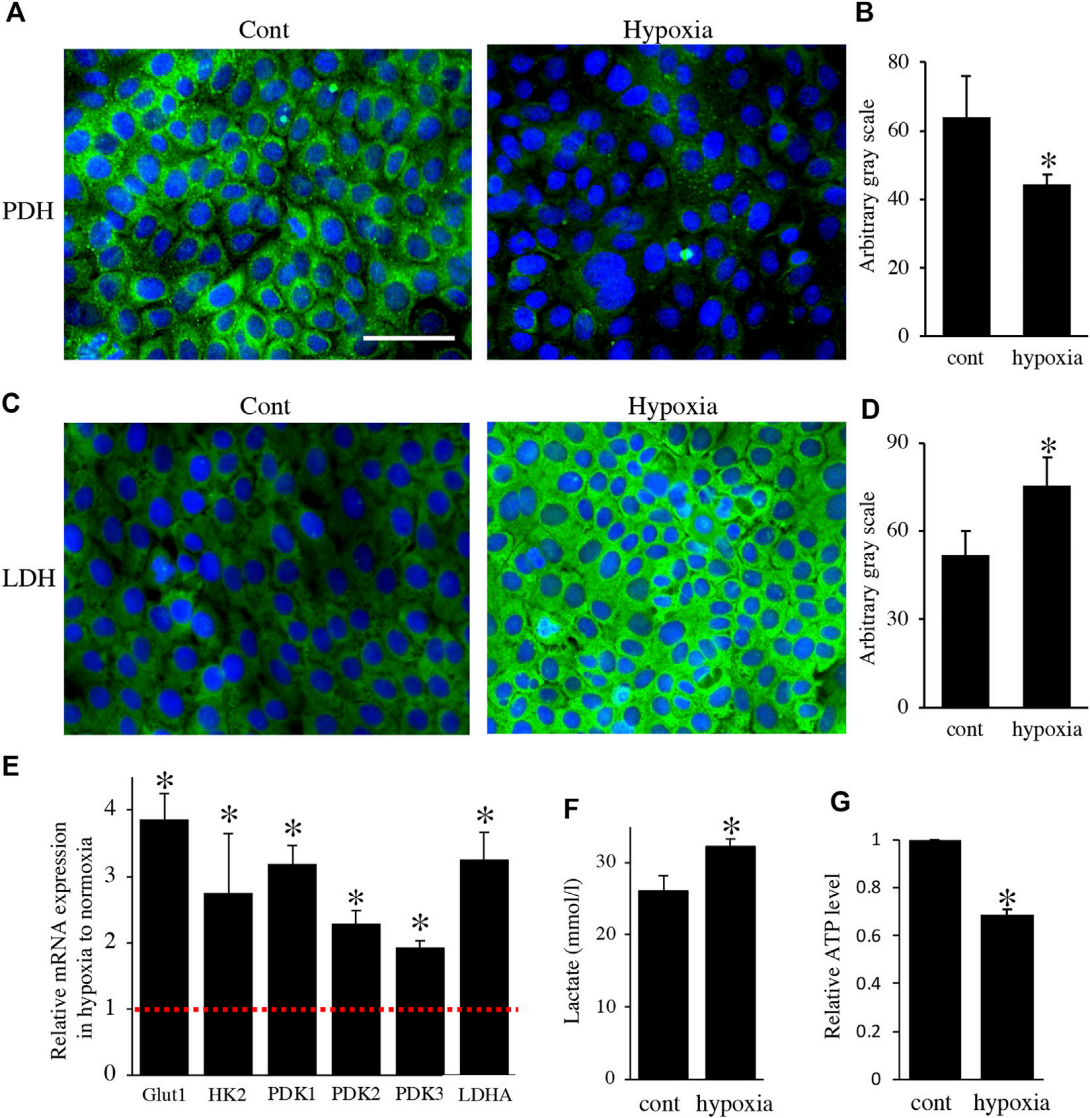
Environmental hypoxia induced energy metabolic shift to glycolysis in HAT7 cells. Immunostaining for PDH **(A)** and LDH **(C)** in HAT7 cells cultured under normoxia (left) and hypoxia (right) for 48 h. Nuclei were stained with DAPI (blue). Quantification of PDH **(B)** and LDH **(D)** fluorescence; *n* = 3 each. **(E)** Relative expression of the target genes in HAT7 cells under hypoxia for 48 h under normoxia; *n* = 3. **(F)** Lactate secretion into the culture medium of HAT7 cells incubated for 48 h under normoxia or hypoxia; *n* = 3. **(G)** Intracellular ATP production in HAT7 cells incubated for 48 h under normoxia or hypoxia (*n* = 3). Data are presented as the mean ± SD. *p < 0.05 (unpaired two-tailed Student’s *t*-test).

**FIGURE 5:**
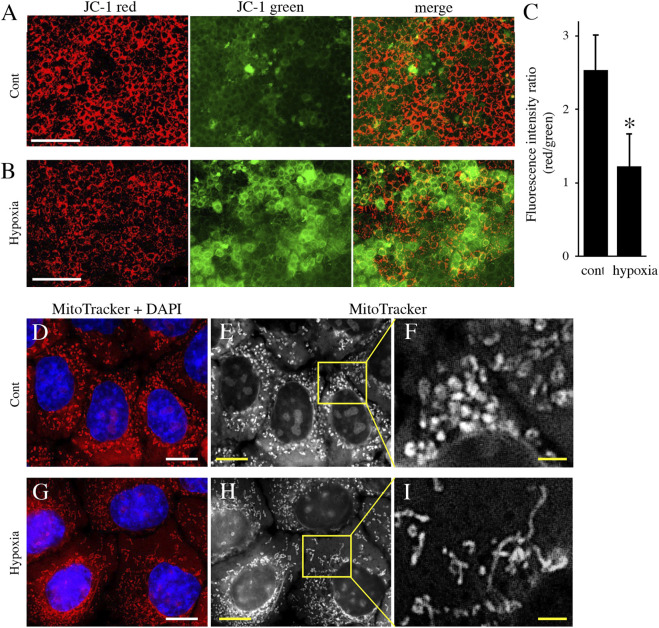
The effect of environmental hypoxia on mitochondrial membrane potential and morphology in HAT7 cells. **(A,B)** HAT7 cells cultured in hypoxia display a decrease in mitochondrial membrane potential is evident by the lack of red JC-1 aggregate (red) accumulation and higher staining for JC-1 green monomers. **(C)** Quantification of red/green JC-1 staining indicative of membrane potential. *n* = 3. MitoTracker Orange CMXRos staining of HAT7 cells cultured in normoxia **(D–F)** and hypoxia **(G–I)** for 48 h. The boxed area in **(E,H)** are magnified in **(F,J)**, respectively. The nucleus is stained with DAPI (blue). Data are represented as their mean ± SD. *p < 0.05 (unpaired two-tailed Student’s *t*-test). Scale bars, 100 μm **(A,B)**; 10 μm **(D,E,G,H)**; 2 μm **(F–I)**.

Next, to analyze the effect of energy metabolic shift on HAT7 cells, we designed experiments to induce an energy metabolic shift by hypoxia. Immunofluorescence revealed that hypoxic culture (5% O_2_ 48 h) decreased PDH expression (Figures 4A,B) and increased LDH expression (Figures 4C,D) possibly without induction of apoptosis (Supplementary Figure S1). qPCR analysis also showed that hypoxia increased the gene expression of glycolytic markers, such as Glut1, Hexokinase 2 (HK2), PDK1, PDK2, PDK3, and LDHA (Figure 4E), and lactate production (Figure 4F), and decreased intracellular ATP production (Figure 4G). We further analyzed the effect of hypoxia on the mitochondrial membrane potential and morphology. JC-1 dye accumulates preferentially in polarized mitochondria, existing as green fluorescent monomers at low membrane potentials and as red fluorescent aggregates at high membrane potentials. Under hypoxia, the red/green fluorescence ratio decreased (Figures 5A–C), indicating depolarization of the mitochondrial membrane potential. Mitochondrial morphology was evaluated using MitoTracker™ Orange CMTMRos. Under normoxia, large mitochondria exhibited a spherical or oval morphology (Figures 5D–F), whereas, under hypoxia, mitochondria exhibited a tubular morphology (Figures 5G–I). These results indicate that HAT7 cells undergo an energy metabolic shift that is dependent on oxygen concentration, accompanied by changes in mitochondrial function and morphology.

### Effect of energy metabolic shift on maturation stage ameloblasts function

**FIGURE 6:**
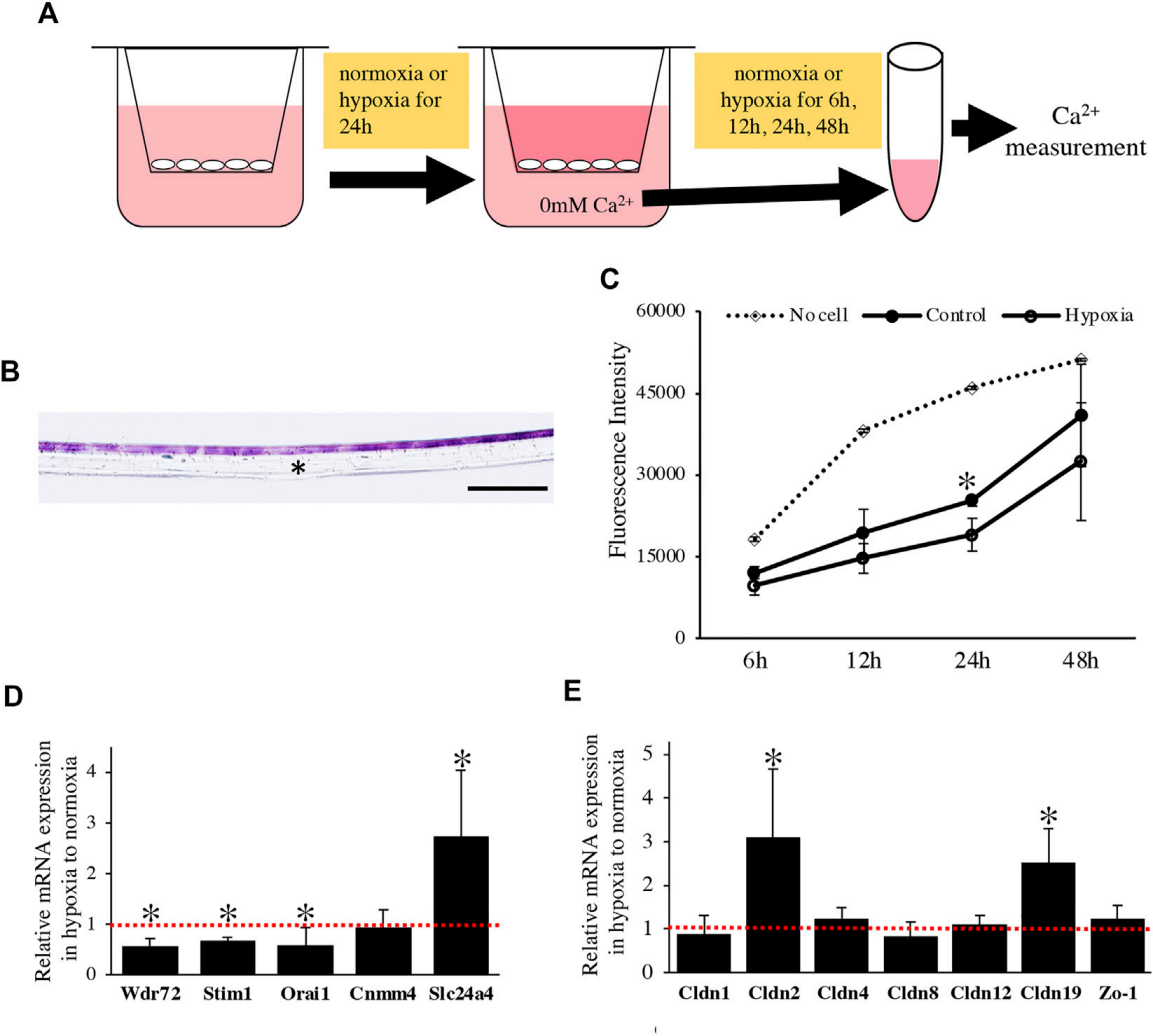
The effect of oxygen-mediated energy metabolic shift on Ca^2+^ transport of HAT7 cells. **(A)** The experimental procedure for *in vitro* calcium transport assay. For more detailed information, see the materials and methods section. **(B)** HE staining of HAT7 cells cultured on Transwell filter in cross-section. **(C)** Changes over time in the amount of calcium in the lower chamber. Calcium was transferred from the upper chamber to the lower chamber through HAT7 cells cultured in normoxia or hypoxia. *n* = 3. **(D,E)** Relative expression of target genes in HAT7 cells under hypoxia for 48 h to normoxia; *n* = 3. Data are represented as their mean ± SD. *p < 0.05 (unpaired two-tailed Student’s *t*-test). Scale bars, 100 μm **(B)**.

We examined the effect of the oxygen-mediated energy metabolic shift on HAT7 cells. During enamel mineralization, calcium is transported from the blood vessels in the papillary layer to the enamel matrix across M-ABs. Therefore, we developed an *in vitro* experimental model to analyze calcium transport across M-ABs in HAT7 cells. The cells were cultured on Transwell culture inserts, reached confluence, and then cultured under normoxia or hypoxia for 24 h. Subsequently, the culture medium in the lower chamber was replaced with Ca^2+^ free medium. The amount of Ca^2+^in the medium of the lower chamber under normoxia or hypoxia was measured using a fluorescent Ca^2+^ probe at each time point (Figure 6A). HE staining of the transverse section after reaching confluence showed that the cells mostly formed a single or 2-cell layer (Figure 6B). We confirmed that the cell layer significantly hindered Ca^2+^ transfer from the upper chamber to the lower chamber compared to the control Transwell surface covered with no cells under normoxia (Figure 6C). Furthermore, hypoxia significantly reduced Ca^2+^ transport (Figure 6C).

We performed a qPCR assay to determine the effect of the oxygen-mediated energy metabolic shift on gene expression related to transcellular and paracellular Ca^2+^ transport. Hypoxia significantly decreased the expression of mRNA related to transcellular Ca^2+^ transport, such as Wdr72, Stim1, and Orai1, and increased Slc24a4 (Figure 6D). Claudin (Cldn) determines the barrier function of tight junctions and creates paracellular pores (channels) for Ca^2+^ between neighboring cells (Günzel and Yu, 2013). In HAT7 cells, hypoxia increased the mRNA expression of Cldn2 and Cldn19, but not that of Cldn1, 4, 8, 12, or Zo-1 (Figure 6E).

**FIGURE 7:**
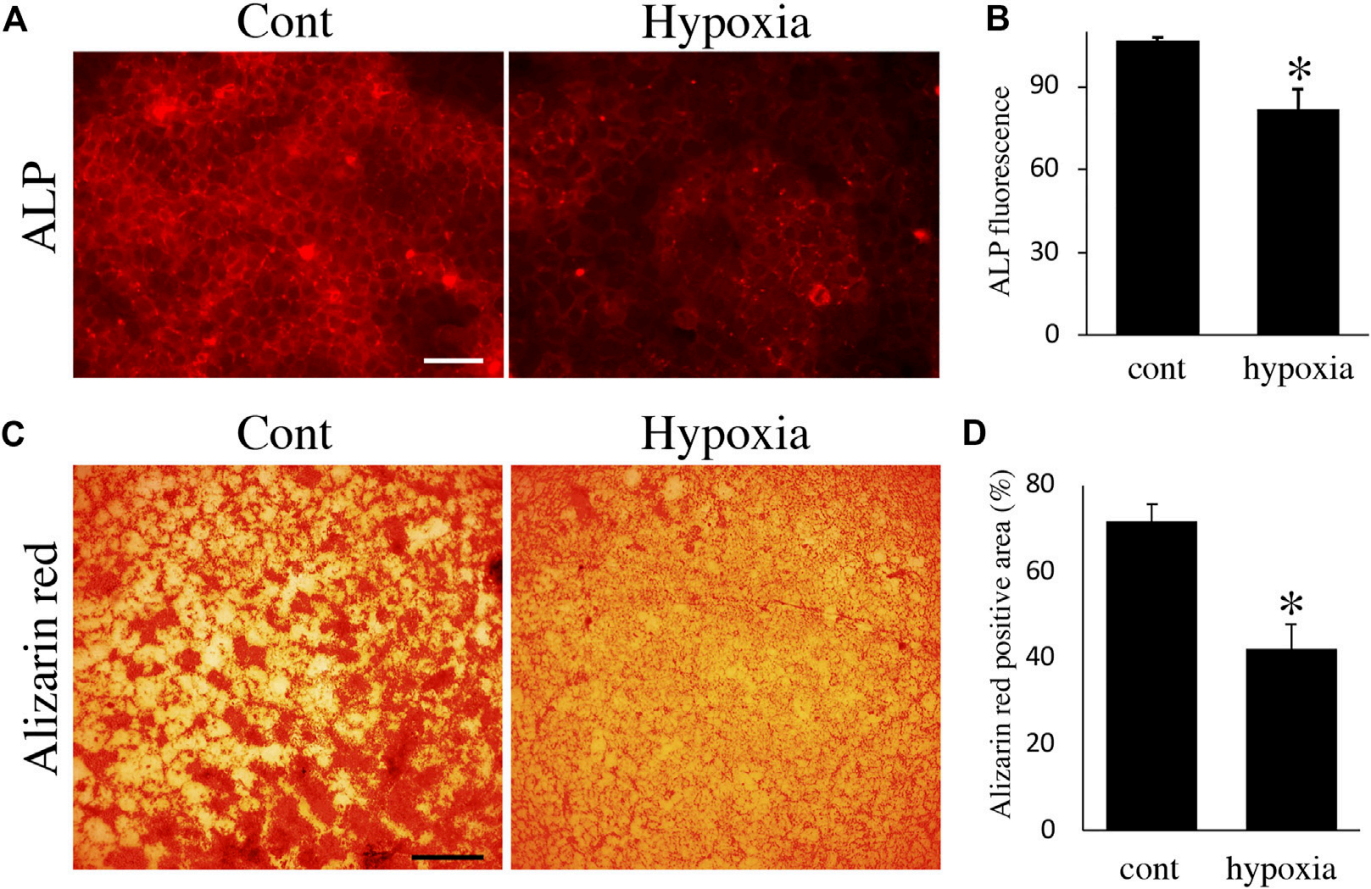
The effect of oxygen-mediated energy metabolic shift on mineralization. **(A)** ALP staining of HAT7 cells cultured in normoxia (left) and hypoxia (right) for 48 h. **(B)** Quantification of ALP fluorescence; *n* = 3. **(C)** Alizarin red staining of HAT7 cells cultured in normoxia (left) and hypoxia (right) for 7 days. **(D)** Image analysis of the mineral coverage (Alizarin red positive) in the culture dish; *n* = 3. Data are represented as their mean ± SD. *p < 0.05 (unpaired two-tailed Student’s *t*-test). Scale bars, 50 μm **(A)**; 500 μm **(C)**.

**FIGURE 8:**
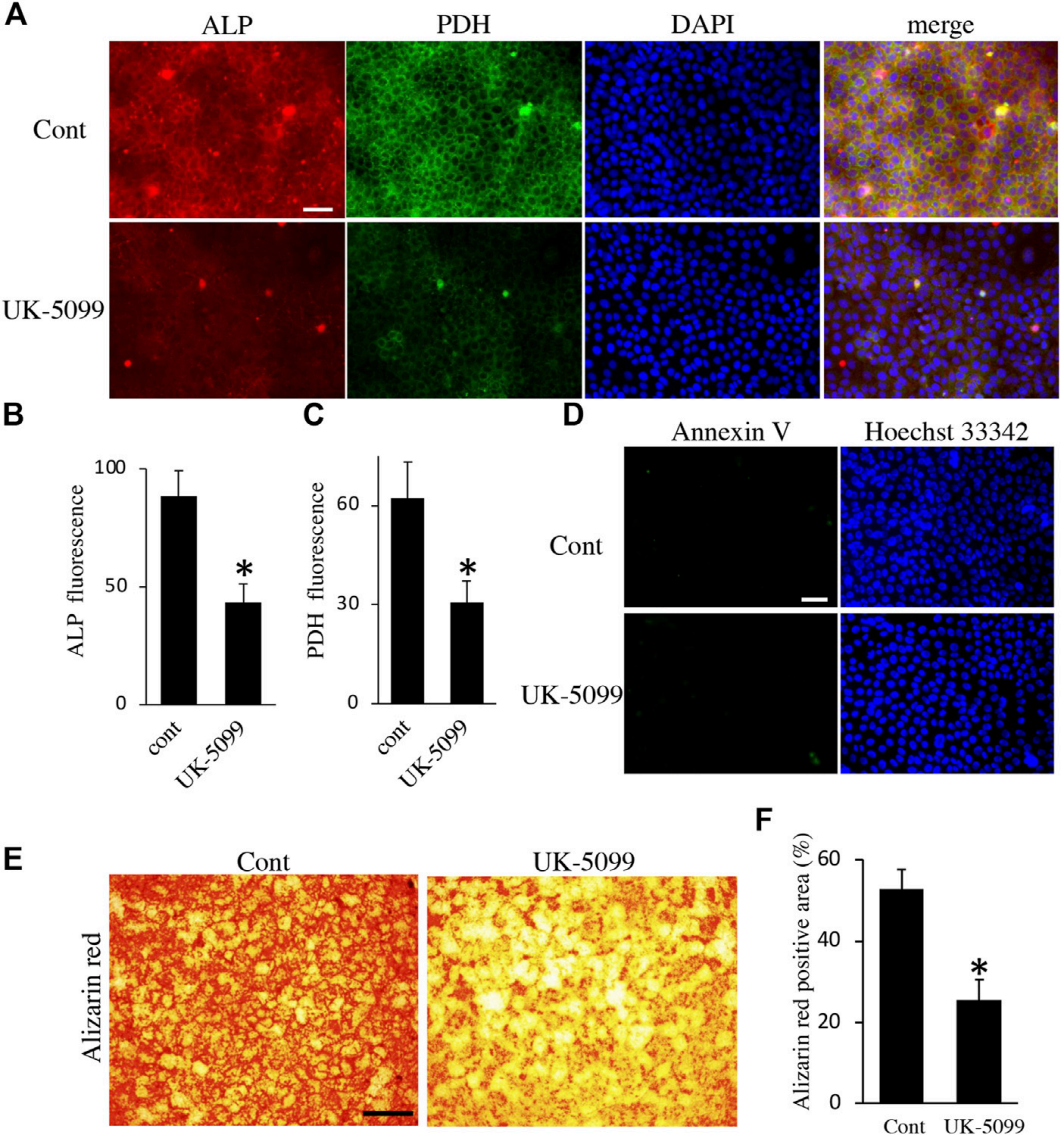
The effect of UK-5099 on mineralization. **(A)** Double staining of ALP and PDH in HAT7 cells treated with DMSO (upper: control) and 10 μM UK-5099 (lower) for 48 h. The nucleus is stained with DAPI (blue). Quantification of ALP **(B)** and PDH **(C)** fluorescence; *n* = 3 each. **(D)** Annexin V staining of HAT7 cells cultured with DMSO (upper: control) and 10 μM UK-5099 (lower) for 48 h. The nucleus is stained with Hoechst 33,342 (blue). **(E)** Alizarin red staining of HAT7 cells cultured with DMSO (left: control) and 10 μM UK-5099 (right) for 5 days. **(F)** Image analysis of the mineral coverage (Alizarin red positive) in the culture dish; *n* = 3. Data are represented as their mean ± SD. *p < 0.05 (unpaired two-tailed Student’s *t*-test). Scale bars, 50 μm **(A,D)**; 500 μm **(E)**.

Finally, we examined the effects of energy metabolic shifts on ALP activity and Ca^2+^ deposition. ALP staining revealed that ALP activity was reduced by hypoxia (Figures 7A,B). Alizarin red staining also showed that hypoxia inhibited Ca^2+^ deposition (Figures 7C,D and Supplementary Figure S2A). Furthermore, UK-5099, an inhibitor of the mitochondrial pyruvate transporter (MPT) that induces energy metabolic shift from OXPHOS to glycolysis (Zhong et al., 2015), significantly decreased ALP activity and PDH expression (Figures 8A–C) possibly without induction of apoptosis (Figure 8D). UK-5099 also inhibited Ca^2+^ deposition (Figures 8E,F and Supplementary Figure S2B).

## Discussion

**FIGURE 9:**
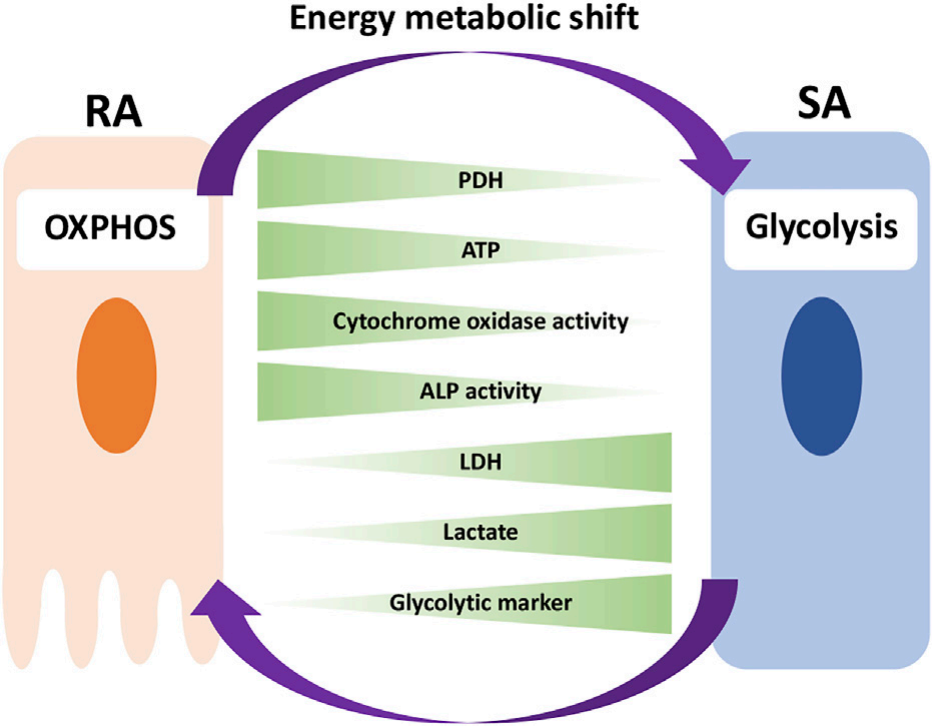
Model for the implication of energy metabolism in RA-SA modulation. RA cells are in an OXPHOS-dominant energy metabolic state, whereas SA cells are in a glycolysis-dominant energy metabolic state. An energy metabolic shift from OXPHOS to glycolysis decreases the mineralization function of M-ABs, implying a phenotypic change from RA to SA.

In this study, we have shown that, *in vivo*, RA cells are in an OXPHOS-dominant energy metabolic state, whereas SA cells are in a glycolysis-dominant energy metabolic state. *In vitro* experiment revealed that an energy metabolic shift from OXPHOS to glycolysis decreased the mineralization function by suppressing ALP activity and Ca^2+^ transport, implying the induction of phenotypic changes from RA to SA. Together, we have identified differences in the energy metabolic properties of RA and SA in M-ABs and highlighted the importance of the energy metabolic state for M-AB regulation (Figure 9).

### A metabolic switch is activated during ameloblast differentiation

We identified the differentiation stages of ameloblasts based on the expression of Zo-1 (Inai et al., 2008) and ALP (Okumura et al., 2010) and examined the expression of metabolic markers in each cell. From S-ABs to RA, the expression of OXPHOS markers increased, whereas that of glycolytic markers decreased. In contrast, from RA to SA in early M-ABs, OXPHOS markers and mitochondrial activity decreased, and glycolytic markers increased. This indicated that a gradual metabolic shift to an OXPHOS-dominant energy metabolism state occurs from S-ABs to RA, and conversely, a shift to a glycolysis-dominant energy metabolism state occurs from RA to SA. In line with this, previous studies have shown that in the transition stage, the expression of many genes involved in ion transport, proteolysis, and pH homeostasis, which required sufficient ATP production, was upregulated (Hu et al., 2012; Lacruz et al., 2012; Wang et al., 2014; Yin et al., 2014). Ultrastructural and cytochemical studies have suggested that in comparison with RA, SA is metabolically inactive and renews exhausted cytoplasmic organelles (Takano and Ozawa, 1980). We also showed that sodium-dependent active glucose transporter 2 (SGLT2), which is expressed in highly metabolically active cells, is expressed in RA but not in SA (Ida-Yonemochi et al., 2020). These results strongly indicated that ameloblasts could shift their metabolic state to meet the cell energy demand for their respective cellular functions, allowing us to identify the differentiation stage of ameloblasts in terms of energy metabolic status.

### Environmental oxygen induces energy metabolic shifts

To analyze the effect of energy metabolic shift on HAT7 cells, we performed experiments to induce an energy metabolic shift by hypoxia. For most cell types, hypoxia has been found to decrease the levels of respiratory enzymes and oxygen consumption rate but increase the production of glycolytic enzymes and lactate, which eventually forces the cells to rely on glycolysis (Kierans and Taylor, 2021). Indeed, in HAT7 cells, hypoxia increases lactate production and the expression of Glut1 (Ebert et al., 1995; Ida-Yonemochi et al., 2020), HK2 (Iyer et al., 1998), PDH (Golias et al., 2016), PDK1-3 (Kim et al., 2006; Lu et al., 2008; Takubo et al., 2013), and LDHA (Yang et al., 2014). Hypoxia alters mitochondrial morphology and function (Galloway et al., 2012). Under hypoxia, the activity of the mitochondrial electron transport chain decreases, and energy needs to shift from OXPHOX to glycolysis (Ježek et al., 2010). We demonstrated that in HAT7 cells, hypoxia changed mitochondrial morphology and reduced ATP production and JC-1 red/green ratio, indicating mitochondrial depolarization and loss-of-function. These results indicated that hypoxia induced an energy metabolic shift in HAT7 cells from OXPHOS-dominant to a more glycolysis-dominant state, implying a phenotypic change from RA to SA.

### Energy metabolic shift affects M-ABs mineralization function

M-ABs are responsible for enamel mineralization through an increase in calcium influx across the ameloblast layer into the enamel matrix. Here, we have shown that an energy metabolic shift alters the enamel mineralization function of M-ABs. We developed a novel *in vitro* experimental model and demonstrated that hypoxia-induced energy metabolic shift to a glycolysis-dominant state reduced Ca^2+^ transport across M-ABs, Ca^2+^ deposition and ALP activity. Further, we demonstrated that UK-5099, that induces energy metabolic shift from OXPHOS to glycolysis, inhibited Ca^2+^ deposition and ALP activity. These findings indicate the critical involvement of energy metabolism in enamel mineralization. Consistent with our findings, Kim et al. demonstrated that hypoxia inhibited normal enamel mineralization in a tooth germ transplantation model (Kim et al., 2021).

### Involvement of energy metabolic shift in trans- and intracellular Ca^2+^ transport in M-ABs

Recent reports suggest that Ca^2+^ transport follows a proximal to distal route across the ameloblast cell layer to form mature enamel crystals. The principal mode of Ca^2+^ transport appears to be the transcellular route (Paine et al., 2008; Lacruz et al., 2013), while the contribution of the paracellular passage of Ca^2+^ during the RA to SA cycles has been indicated (Smith, 1979; Nanci, 2008). In the present study, we showed that oxygen-mediated energy metabolic shifts affected the expression of genes involved in both trans- and paracellular Ca^2+^ transport. We showed that hypoxia decreased the expression of Orai1 and Stim1. When Stim1 senses a decrease in Ca^2+^ in the endoplasmic reticulum, it forms clusters in the proximal region of the ER and plasma membrane and activates Orai1, which triggers store-operated Ca^2+^ entry (SOCE) (Prakriya and Lewis, 2015). In M-ABs, SOCE *via* the Orai1-Stim1 complex has been suggested to be the main calcium influx pathway (Nurbaeva et al., 2017), and patients with loss-of-function or null mutations in the STIM1 and ORAI1 genes present with a hypocalcified form of amelogenesis imperfecta (Mccarl et al., 2009; Picard et al., 2009; Fuchs et al., 2012). Thus, an energy metabolic shift may have a significant effect on transcellular calcium transport *via* the Orai1-Stim1 complex in M-ABs. In addition, Orai1 and Stim1 were reported to be predominantly expressed in RA compared to SA (Nurbaeva et al., 2015; Nurbaeva et al., 2017), indicating that Ca^2+^ uptake may predominantly occur in RA, which requires more oxygen for energy production than SA. Therefore, hypoxia may have a greater effect on RA function than on SA.

Furthermore, hypoxia reduced WDR72 expression. Mutation of the WDR72 gene results in hypomaturation defects of the enamel, which are thought to be caused by the abnormal removal of enamel matrix proteins and subsequent enamel mineralization (Katsura et al., 2014; Wang et al., 2015). Mutations in WDR72 have also been shown to decrease the number and size of blood vessels in the capillary layer and alter the subcellular localization of SLC24a4 (sodium/potassium/calcium exchanger 4; NCKX4), which is critical for transcellular Ca^2+^ transport in M-ABs (Wang et al., 2015). Interestingly, our data showed that hypoxia increased SLC24a4 mRNA expression. We speculated that this may have occurred to compensate for the mislocalization of Slc24a4 caused by the decrease in WDR72.

Paracellular access of ions and small molecules to form enamel depends on the composition of TJs, including members of the zonula occludens, occludin, and claudin families (Denker and Sabath, 2011). A combination of different claudins either allows intercellular passage of ions or is tightly closed and restricts passage (Günzel and Yu, 2013). We demonstrated that hypoxia decreased Ca^2+^ transport across HAT7 cells with an increase in CLND2 and 19 mRNA expression. This result suggests that CLDN2 and 19 may contribute to inhibit paracellular Ca^2+^ transport in M-ABs. CLDN2 has been identified as a cation pore-forming protein (Günzel and Yu, 2013). In the renal proximal tubule, TJs containing CLDN 2 have been shown to be leaky and have low transepithelial resistance (Denker and Sabath, 2011). Recently, a missense mutation in Cldn2 associated with obstructive azoospermia in a four-generation spanning family has been identified (Seker et al., 2019). Cldn2 KO mice have also shown higher urinary fractional excretion of Ca^2+^ in renal proximal tubules (Muto et al., 2010). However, the function of CLDN2 in the ameloblasts remains unclear. Cldn19 has been shown to be located in tight junctions of ameloblasts in mice and rats, where it plays a role in regulating extracellular pH, which is critical for the processing and secretion of extracellular matrix proteins (Bardet et al., 2017; Yamaguti et al., 2017). Mutations in CLDN19 are associated with amelogenesis imperfecta, a genetic disorder characterized by tooth enamel defects (Bardet et al., 2017; Yamaguti et al., 2017). These reports indicate that CLDN19 plays a critical role in amelogenesis. However, the detailed involvement of paracellular Ca^2+^ transport in M-ABs is unknown. Thus, further investigation of the role of each CLDN isoform in paracellular Ca^2+^ transport in M-ABs is required.

Although we used HAT7 cells to clarify the implication of an energy metabolic shift in M-ABs, we must note the limitation of the model. HAT7 cells are established from rat ameloblasts and express M-ABs markers, but alone cannot be a sufficient model for M-ABs. Besides Ca^2+^ transport and mineralization, additional mechanisms have to be identified, such as morphological change and protein degradation and absorption, as well as their coordinating mechanism. Thus, more complex cell culture models and analysis methods need to be developed in the future for better modeling of M-ABs. In addition, the oxygen concentration of M-ABs *in vivo* is different from *in vitro* conditions. Therefore, direct measurements of oxygen concentration *in vivo* and animal experiments under hypoxic conditions will help to identify the correlation between oxygen concentration and energy metabolism in M-ABs, and elucidate the regulatory mechanisms underlying RA-SA modulation.

### Contribution and importance of this research in clinical dental medicine

In this study, we uncovered the energy metabolic characteristics of ameloblasts and demonstrated the involvement of energy metabolic shifts in the phenotype modulation of M-ABs. This discovery not only has a significant impact on our understanding of the regulatory mechanism underlying normal amelogenesis but also raises the possibility that failure of this mechanism can cause enamel malformation in human patients. To date, a variety of causal genes for inherited enamel malformations have been identified. These genes are involved in diverse functions, such as the secretion of enamel matrix proteins and their proteolytic processing enzymes, vesicle transport, pH sensing, calcium homeostasis, and cell adhesion (Smith et al., 2017). However, the involvement of energy metabolism in enamel malformation has not been demonstrated. Intriguingly, it was recently suggested that more common enamel defects, such as molar incisor hypomineralization (MIH), defined as a qualitative, demarcated, enamel defect of hypomineralization affecting at least one first permanent molar, while permanent incisors are often affected (Weerheijm et al., 2001), were caused by perinatal hypoxia (Garot et al., 2022). Therefore, further studies to clarify whether the abnormality of energy metabolic regulation causes enamel defects by interacting with intracellular signal networks and environmental factors in humans will aid in the development of novel treatment and prevention strategies for enamel malformations.

## Data Availability

Datasets are available on request: The raw data supporting the conclusions of this article will be made available by the authors, without undue reservation.

## Supplementary material

SUPPLEMENTARY FIGURE S1
Annexin V staining of HAT7 cells cultured in hypoxia. **(A)** Double staining of Hoechst 33342 (left) and Annexin V (middle) in HAT7 cells cultured on plastic plates under normoxia (upper: control) and hypoxia (lower) for 48 h. **(B)** Double staining of Hoechst 33342 (left) and Annexin V (middle) in HAT7 cells treated with 50 μM mitomycin C for 6 h. Scale bars, 50 μm.

SUPPLEMENTARY FIGURE S2
DAPI staining of HAT7 cells cultured on plastic plates coated with collagen type I for alizarin red staining. **(A)** DAPI staining of HAT7 cells cultured under normoxia (left) and hypoxia (right) for 7 days. **(B)** DAPI staining of HAT7 cells cultured with DMSO (left: control) and UK-5099 (right) for 5 days. Scale bars, 200 μm.
