# Supplementary figures and images for: Energy metabolic shift contributes to the phenotype modulation of maturation stage ameloblasts
Source: Front Physiol. 2022 Nov 29;13:1062042. doi: 10.3389/fphys.2022.1062042 (PMC9745043; doi:10.3389/fphys.2022.1062042)

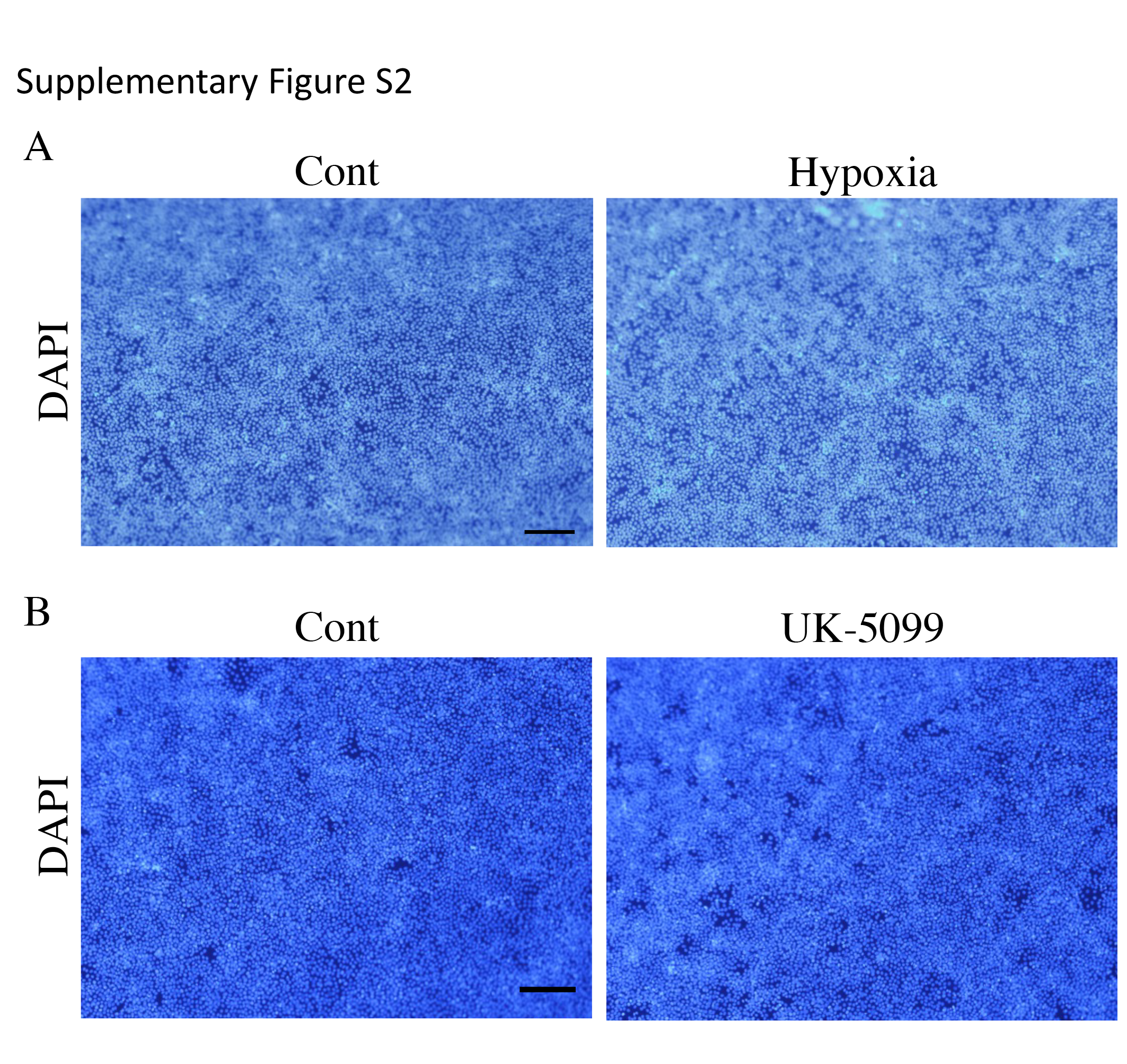

Supplement: Supplementary file 1 [file Image2.TIF]

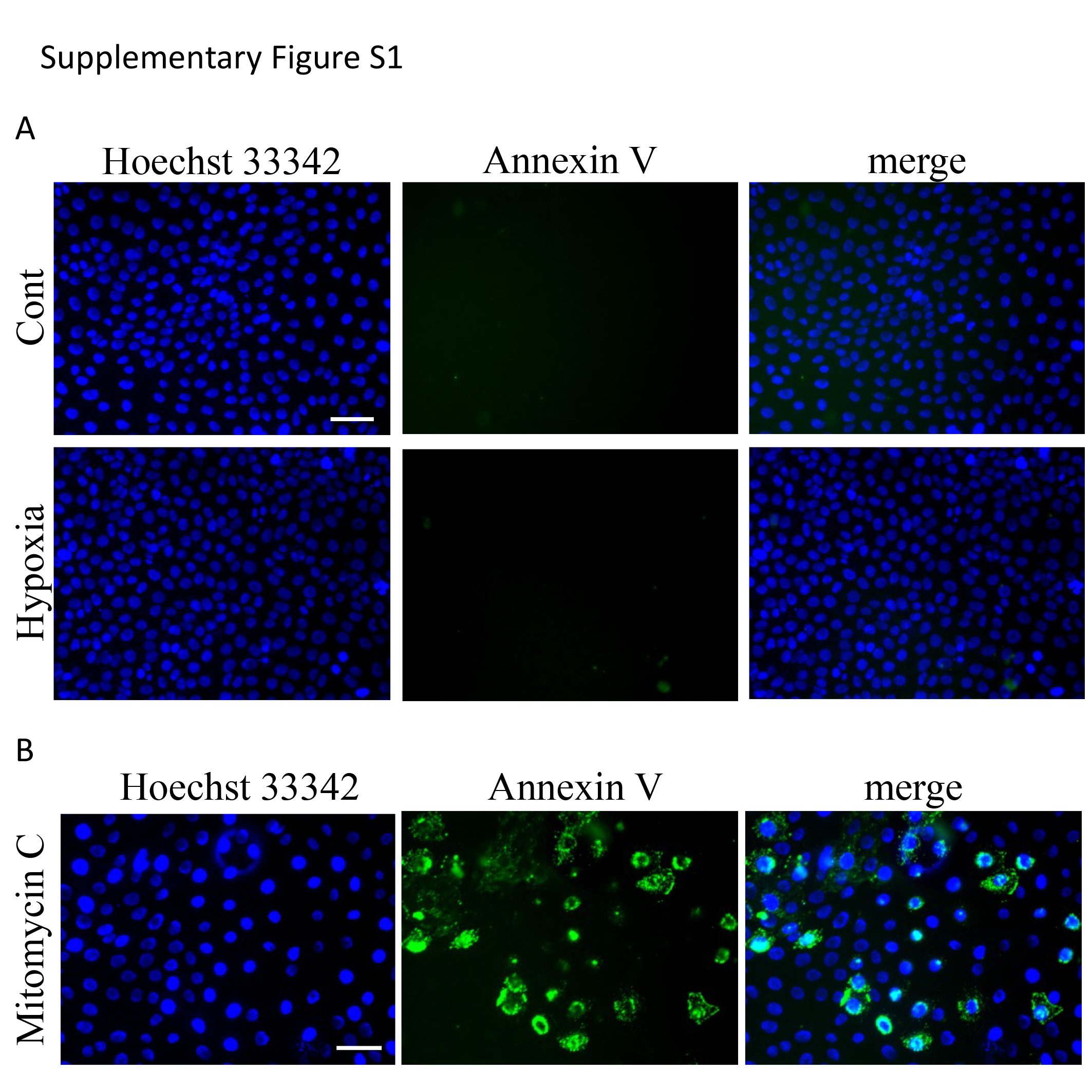

Supplement: Supplementary file 2 [file Image1.TIF]
